# Supplementary material for: In exogenous attention, time is the clue: Brain and heart interactions to survive threatening stimuli
Source: PLoS One. 2021 May 12;16(5):e0243117. doi: 10.1371/journal.pone.0243117 (PMC8115771; doi:10.1371/journal.pone.0243117)
Supplement: S1 File — (DOCX) [file pone.0243117.s001.docx]

**S1 File. Results on the relationship between Trait anxiety and Disgust sensitivity and exogenous attention to emotional stimuli**

Trait anxiety and disgust sensitivity (DS) of all participants were evaluated through the State-Trait Anxiety Inventory (STAI; [1], Spanish adaptation by [2]) and the Disgust Scale-Revised (DS-R; [3], modified by [4]; Spanish version by [5]), respectively, although data from three participants were missing.

The results of the analysis failed to show any main effect of trait anxiety or disgust sensitivity for RTs, error rates, P1, P2, or N2. The only interaction that was statistically significant was the emotional distractor by DS scores for the P1 amplitude (F(1.94, 48.53) = 3.196, p = .051, η^2^ = .113). The analysis of this interaction revealed that the high DS group showed the pattern of response described for the whole sample in the results section, where P1 amplitudes were higher for F stimuli compared to N and D stimuli (p < .02). The low DS group showed higher P1 amplitudes to F and D stimuli compared to N, although only the response to F stimuli reached significance (p = .026).

**References**

1. Spielberger CD, Gorsuch RL, Lushene RE. Manual for the state-trait inventory. Palo Alto, CA: Consulting Psychologists Press; 1970.

2. Seisdedos N. Adaptación Española del STAI, Cuestionario de ansiedad estado-rasgo Spanish adaptation of the STAI, State-Trait Anxiety Inventory. Madrid: Tea Ediciones; 1988.

3. Haidt J, McCauley C, Rozin P. Individual differences in sensitivity to disgust: a scale sampling seven domains of disgust elicitors. Pers Individ Differ. 1994; 16: 701–713.

4. Olatunji BO, Williams NL, Tolin DF, Sawchuck CN, Abramowitz JS, Lohr JM, et al. The disgust scale: item analysis, factor structure, and suggestions for refinement. Psychol Assess. 2007; 19: 281–297.

5. Sandín B, Valiente RM, Chorot P. Instrumentos para la evaluación de los miedos y las fobias. In Sandín B, editor. Las fobias específicas. Madrid: Klinik; 2008.
