# Supplementary material for: In exogenous attention, time is the clue: Brain and heart interactions to survive threatening stimuli
Source: PLoS One. 2021 May 12;16(5):e0243117. doi: 10.1371/journal.pone.0243117 (PMC8115771; doi:10.1371/journal.pone.0243117)
Supplement: S2 File — (DOCX) [file pone.0243117.s002.docx]

**S2 File. Analyses for checking statistical power.**

Statistical power was computed in two ways. Whereas the observed power for the ANOVAs was computed using SPSS, the post-hoc power of repeated-measure ANOVAs was estimated by means G*Power [1]. The observed power for the interaction effect between the Distractor x HRV group for each of ERP components is provided below:

-P1-SF1: F(1.97,55.26) = 5.729, p = .006; eta^2^= 0.170; (1- β = 0.9976).

-P1-SF2: F(1.95,54.65) = 3.429, p = .041; eta^2^= 0.109; (1- β = 0.9588).

-P2-SF2: F(1.99,55.696) = 3.710, p = .031; eta^2^= 0.117; (1- β = 0.9704).

-N2-SF1: F(1.97,55.18) = 5.462, p = .007; eta^2^= 0.163; (1- β = 0.9965).

-N2-SF2: F(1.9,53.27) = 4.225, p = .021; eta^2^= 0.131; (1- β = 0.9839).

-N2-SF3: F(1.89,52.90) = 6.125, p = .005; eta^2^= 0.179]; (1- β = 0.9985).

Reference

1. Faul F, Erdfelder E, Lang AG, Buchner A. G*Power 3: A flexible statistical power analysis program for the social, behavioral, and biomedical sciences. Behav Res Methods. 2007 39:175–191. doi:10.3758/BF03193146
